# Quantitative Evaluation of Changes in Retinal and Choroidal Blood Flow Following Strabismus Surgery

**DOI:** 10.1167/tvst.14.3.12

**Published:** 2025-03-12

**Authors:** Sayuri Yasuda, Yoshiko Takai, Yuma Yasuda, Takanori Yamamoto, Ryo Tomita, Takeshi Iwase, Norie Nonobe, Jun Takeuchi, Taiki Kojima, Koji M. Nishiguchi, Hiroki Kaneko

**Affiliations:** 1 Department of Ophthalmology, Nagoya University Graduate School of Medicine, Nagoya, Japan; 2 Department of Emergency and Critical Medicine, Nagoya University Graduate School of Medicine, Nagoya, Japan; 3 Department of Ophthalmology, Akita University Graduate School of Medicine, Akita, Japan; 4 Ito Eye Clinic, Ichinomiya, Japan; 5 Department of Ophthalmology, Kyorin University School of Medicine, Mitaka, Japan; 6 Department of Anesthesiology, Aichi Children's Health and Medical Center, Obu, Japan; 7 Division of Comprehensive Pediatric Medicine, Nagoya University Graduate School of Medicine, Nagoya, Japan; 8 Department of Ophthalmology, Hamamatsu University School of Medicine, Hamamatsu, Japan

**Keywords:** strabismus surgery, extraocular muscle recession, ocular blood flow, optical coherence tomography angiography, laser speckle flowgraphy

## Abstract

**Purpose:**

This study aimed to investigate the effects of extraocular muscle recession performed as part of strabismus surgery on posterior retinal and choroidal blood flow.

**Methods:**

A single-center prospective study was conducted on patients who underwent strabismus surgery. Optical coherence tomography, optical coherence tomography angiography, and laser speckle flowgraphy of the macula were performed before surgery and at one week, one month, and four months after surgery. Preoperative and postoperative ratios were calculated, and longitudinal changes in retinal blood flow, choroidal thickness, and choroidal blood flow were analyzed. Furthermore, the changes based on the types of resected muscle were examined.

**Results:**

In total, 254 eyes from 127 patients were included. The subfoveal choroidal thickness increased significantly at one week and one month after surgery, with no significant change at four months after surgery. The choroidal blood flow increased significantly at one week after surgery, with no significant changes at one and four months after surgery. The retinal vessel density significantly decreased at one week after surgery, with no significant changes at one and four months after surgery. Analysis of groups that had various muscles excised showed no significant changes in any measurements. Choroidal thickness and blood flow were significantly correlated at one week after surgery.

**Conclusions:**

Strabismus surgery decreased retinal blood flow but increased choroidal thickness and blood flow in the early postoperative period. Moreover, no significant changes were observed in the long term compared to the preoperative period.

**Translational Relevance:**

Strabismus surgery affects the retina and choroid in the early postoperative period but not in the long term.

## Introduction

Strabismus surgery is performed to treat strabismus or anomalous eye positions, and nystagmus. During strabismus surgery, the anterior ciliary artery that is the main blood supply to the anterior segment of the eye is often cut off with the rectus muscle. Hence, although rare, anterior segment ischemia is a potential adverse effect of strabismus surgery.^1^ Simultaneous surgery on multiple rectus muscles is associated with an increased risk of anterior segment ischemia.^2^ Thus several studies have reported that at least one rectus muscle should be left intact in each eye during strabismus surgery.^3^^,^^4^

Some studies have focused on the pathogenesis of anterior segment ischemia and anterior segment blood flow changes after strabismus surgery.^5^^–^^7^ Nevertheless, only a few studies have examined the effects of strabismus surgery on posterior ocular blood flow. The choroid is commonly nourished by the short posterior ciliary arteries. However, the anterior ciliary arteries also contribute to some blood flow. Therefore, strabismus surgery with extraocular muscle resection may decrease choroidal and anterior ocular blood flow. Some reports have investigated posterior ocular blood flow.^8^^–^^11^ However, a consensus regarding the effect of strabismus surgery on choroidal blood flow has not been reached. The choroid is responsible for the blood supply to the retina and sclera, and it provides nutrition and facilitates thermoregulation, and, particularly, retinal oxygenation.^12^ Hence, whether strabismus surgery will affect choroidal blood flow is an important clinical question.

The effects of strabismus surgery on posterior ocular blood flow are challenging to evaluate. One of the reasons why is that it is not easy to quantitatively assess posterior ocular blood flow. In recent years, with the development of novel ophthalmic techniques such as optical coherence tomography angiography (OCTA) and laser speckle flowgraphy (LSFG), it has become possible to more easily and quantitatively assess the retinal and choroidal circulation. OCTA is a novel and noninvasive imaging modality that was recently developed based on optical coherence tomography (OCT). Furthermore, it can be used to visualize the retinal vasculature and the choroidal vasculature, to some extent, without the need for dye injection.^13^ LSFG is a noninvasive method for measuring relative blood flow in real time. To the best of our knowledge, no studies have used LSFG in strabismus surgery. However, several reports have revealed the use of LSFG for evaluating changes in choroidal blood flow after scleral buckling surgery.^14^^–^^16^

The current study aimed to investigate the effects of extraocular muscle surgery on posterior ocular blood flow, as measured on OCTA and LSFG. We hypothesized that strabismus surgery with extraocular muscle recession can reduce both posterior and anterior ocular blood flow.

## Methods

### Participants

This prospective, single-center study included patients who underwent strabismus surgery at Nagoya University Hospital from January 2019 to November 2022. The study protocol was in accordance with the tenets of the Declaration of Helsinki. It was approved by the Ethics Committee of Nagoya University Hospital (2019–0072), Nagoya, Japan, and registered in the University Hospital Medical Information Network Clinical Trials Registry (ID: UMIN000035995). Informed consent or assent was obtained from the patients themselves or the patients’ parents prior to study participation.

Patients <3 years of age were excluded from this study. Additionally, patients with other conditions that interfere with accurate examination, such as nystagmus and amblyopia, those with a history of retinal or choroidal disease, those with a history of internal eye surgery and strabismus surgery, and those who underwent transposition surgery for rectus muscle palsy were excluded from the analysis.

### Study Examination

The patients underwent anterior eye and fundus examinations before strabismus surgery. The refractive error of children was measured with cycloplegia, and the patients also underwent comprehensive ophthalmologic examinations, including best-corrected visual acuity and intraocular pressure measurements. The participants were instructed to avoid drinking alcoholic and caffeinated beverages on the morning of the examinations. To decrease the effect of diurnal fluctuations, all assessments were conducted while the participants were in the sitting position between 10:00 AM and 12:00 PM.^17^^,^^18^ Orthoptic evaluation, OCT (Spectralis; Heidelberg Engineering, Heidelberg, Germany), OCTA (AngioPlex, CIRRUS HD-OCT model 5000; Carl Zeiss Meditec AG, Oberkochen, Germany), and LSFG (LSFG-NAVI; Softcare Co., Ltd., Fukutsu, Japan) were performed before strabismus surgery and at one week, one month, and four months after the surgery. In addition, blood pressure and heart rate were also measured before and after the surgery using an automated blood pressure monitor (CH-483C; Citizen, Tokyo, Japan).

Subfoveal choroidal thickness (SFCT) was measured from the highly reflective zone of the retinal pigment epithelium to the boundary between the choroid and sclera using enhanced-depth imaging OCT, as reported in previous studies.^19^^,^^20^ A radial line scan through the center of the fovea was obtained at an angle of 30°, and we acquired 50 OCT images, which were averaged to reduce speckle noise. Two researchers measured the SFCT. Each researcher conducted six measurements, and the average value was calculated and used for analysis. Retinal blood flow was measured using OCTA. OCTA uses an algorithm called the OCT microangiography complex to generate images of en face microvascular flow using differences in phase and intensity information from successive B-scans performed at the same location.^21^ Using the manufacturer's software, the vessel density (VD) (defined as the area occupied by the blood vessels within a particular area measured in mm^2^/mm^2^), which is an indicator of retinal blood flow, could be determined. In this study, the VD within a 3 × 3 mm circle in the center of the macula was examined. Choroidal blood flow was measured using LSFG. The principles of LSFG have been described in detail elsewhere.^22^^,^^23^ LSFG can detect the speckle contrast pattern caused by the interference of illuminating laser light scattered by the movement of erythrocytes in a blood vessel, and can measure the relative blood flow in the vessel expressed by the mean blur rate (MBR). The MBR of the macula mainly originates from the choroid.^24^ Thus the relative choroidal blood flow can be determined by measuring the MBR of the macula. In this study, we set the center of the square to the macula (250 × 250 pixels, power: 6.31° × 6.31°) and measured the MBR. LSFG was performed three times at each time point in all eyes, and the mean value of the variables was calculated.

### Surgical Technique

Recession or plication of the rectus muscles, recession of the inferior oblique muscle (IO), and tendon lengthening of the superior oblique muscle (SO) were performed. The patients underwent surgeries, which were performed by two surgeons (Y.T. and S.Y.) using the same procedure, using 6-0 Vicryl (Ethicon, Somerville, NJ, USA) for muscle suturing in recession or plication, 5-0 Mersilene (Ethicon) for SO tendon lengthening, and 8-0 Vicryl for conjunctival suturing. The patients received postoperative antimicrobial and steroid eye drops for one month.

### Statistical Analyses

For the summary statistics, categorical variables were expressed as numbers and percentages. Moreover, continuous variables were presented as medians and interquartile ranges as these values had a non-normal distribution. The normality of the continuous variables was tested using the Shapiro-Wilk test or was assessed visually with the Q-Q plot. The preoperative and postoperative (one week, one month, and four months) SFCT, VD, and MBR values were compared using the Wilcoxon signed-rank test (matched-paired comparisons) because these date, which had a non-normal distribution, were longitudinal at the four timepoints in the same personnel. All statistical analyses were performed using JMP 17.2.0 (SAS Institute, Cary, NC, USA) and STATA 18.0 (StataCorp, College Station, TX, USA). For each analysis, the null hypothesis was assessed at a two-tailed significance level of 0.05. To address the issue of type 1 error inflation via multiple pairwise comparisons in the three family-wise groups (SFCT, VD, and MBR), Bonferroni correction was used to adjust the *P* value thresholds. A two-sided *P* value = 0.016 was used as the criterion for statistical significance in the multiple pair-wise comparisons (preoperative vs. one week, one month, and four months after surgery) in the three groups (SFCT, VD, and MBR).

To compare the preoperative and postoperative state of the choroidal thickness and retinal and choroidal blood flow, the postoperative value–to–preoperative value (post-/pre-) ratio was calculated. The post-/pre-ratio was used to perform intragroup comparisons. In addition, because the changes after strabismus surgery may differ based on the surgical procedure, the preoperative and postoperative changes in the retina and choroid in each procedure were examined.

## Results

**Figure 1.**
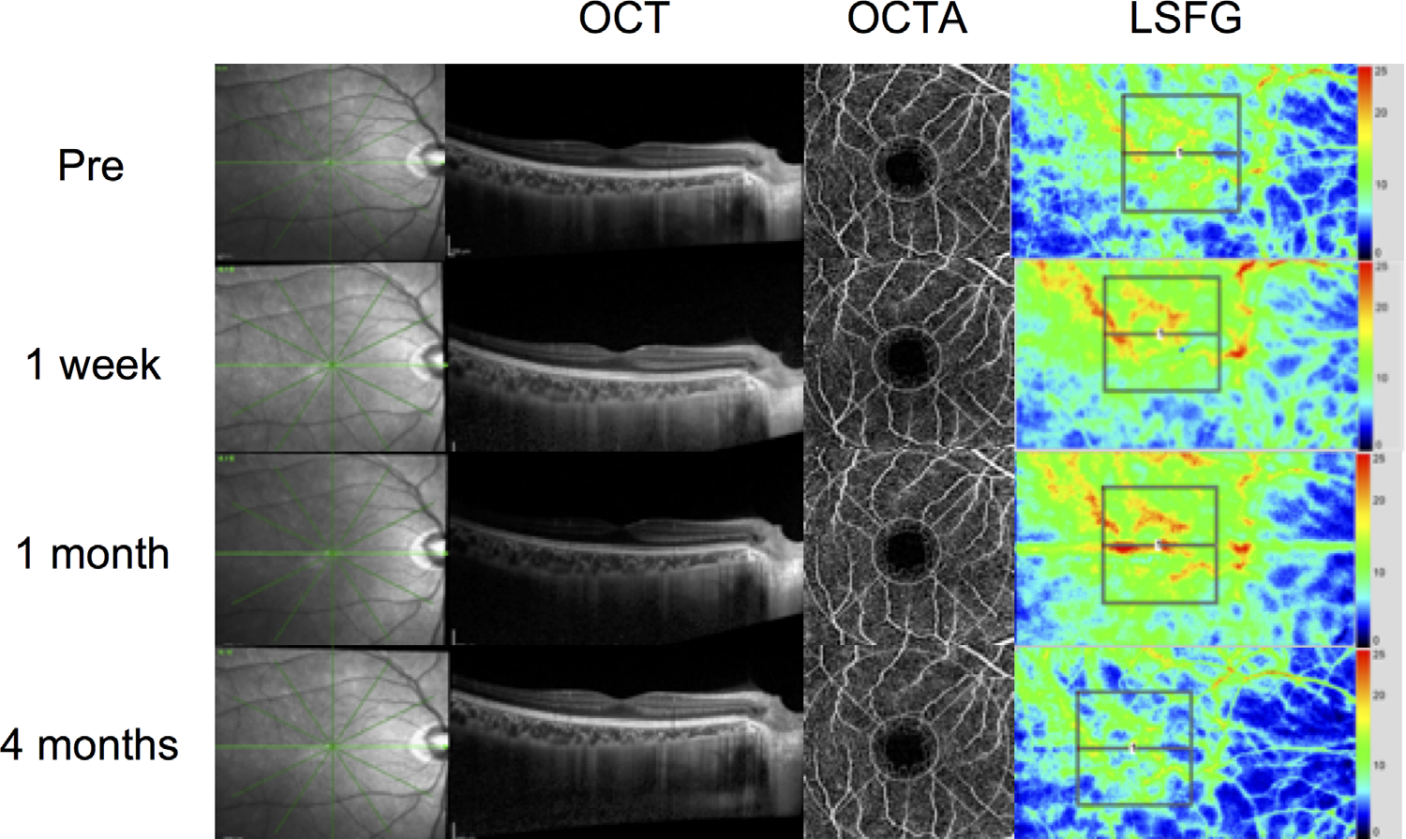
Representative multimodal images of a patient who underwent LR recession and MR plication. Representative macula image of enhanced-depth imaging OCT, OCTA, and synthetic color map of MBR measured via LSFG before and after strabismus surgery with external rectus muscle recession in a 19-year-old male patient who underwent LR recession and MR plication. The subfoveal choroidal thickness increased at one week and one month after surgery. Then, it decreased to the preoperative level at four months after surgery. The vessel density decreased at one week after surgery and then reached the preoperative level. The *red color* indicates a high MBR, and the *blue color* indicates a low MBR. To measure the choroidal MBR, the center of the square was set at the macula (250  × 250 pixels, degree: 6.31°  × 6.31°). The MBR at four months after surgery was lower than that before surgery. LR, lateral rectus muscle; MR, medial rectus muscle; Pre, preoperative; 1 week, one week after surgeries; 1 month, one month after surgeries; 4 months, four months after surgeries.

**Figure 2.**
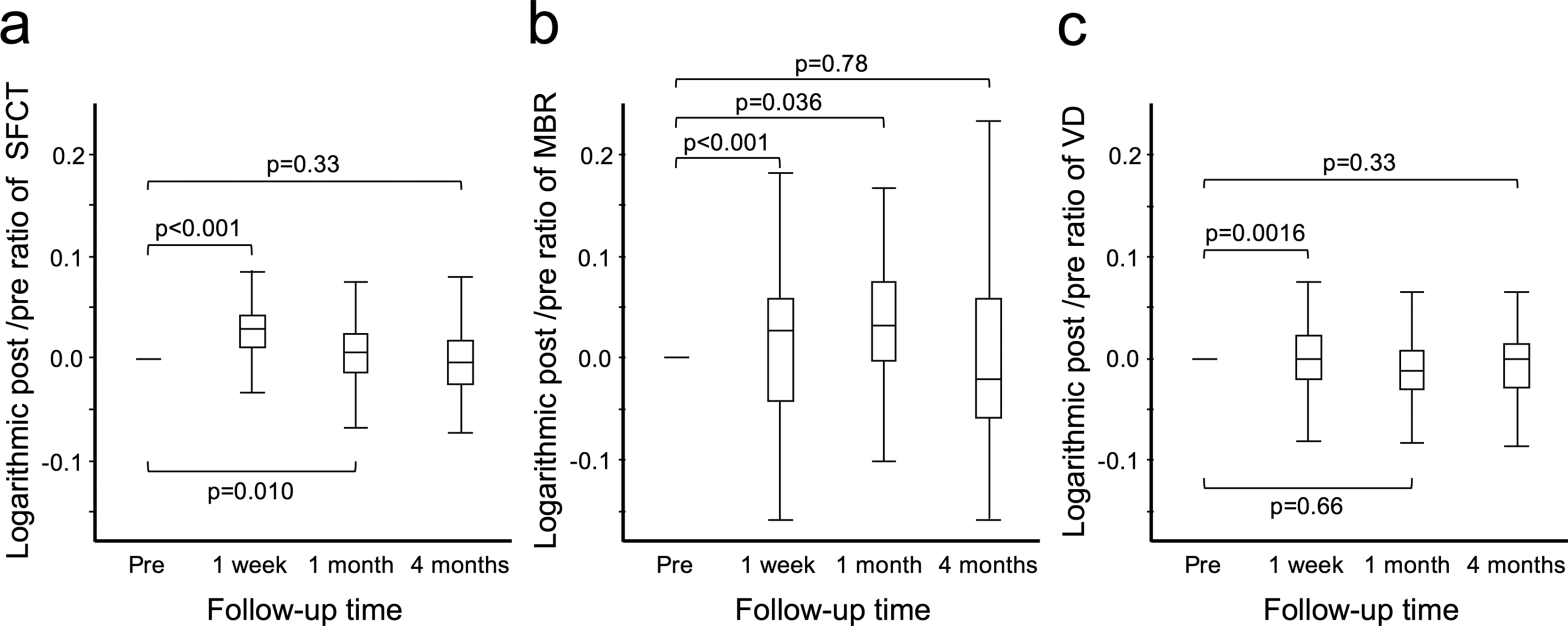
Changes in the post-/pre-ratio of SFCT, MBR, and VD in the eyes undergoing strabismus surgery. (**a**) The SFCT increased significantly at one week and one month after surgery compared with before surgery (*P* < 0.001, *P* = 0.010). However, there was no significant change in the SFCT at four months after surgery (*P* = 0.33) (n = 87). (**b**) The MBR increased significantly at one week after surgery compared with before surgery (*P* < 0.001). Nevertheless, there was no significant change in the MBR at one month and four months after surgery (*P* = 0.036, *P* = 0.78) (n = 70). (**c**) The VD decreased significantly at one week after surgery compared with before surgery (*P* = 0.0016), but there was no significant change in the VD at one and four months after surgery (*P* = 0.66, *P* = 0.33) (n = 94). We only analyzed data that were available from the period before strabismus surgery to four months after surgery. Pre, preoperative, 1 week, one week after surgeries; 1 month, one month after surgeries; 4 months, four months after surgeries.

**Figure 3.**
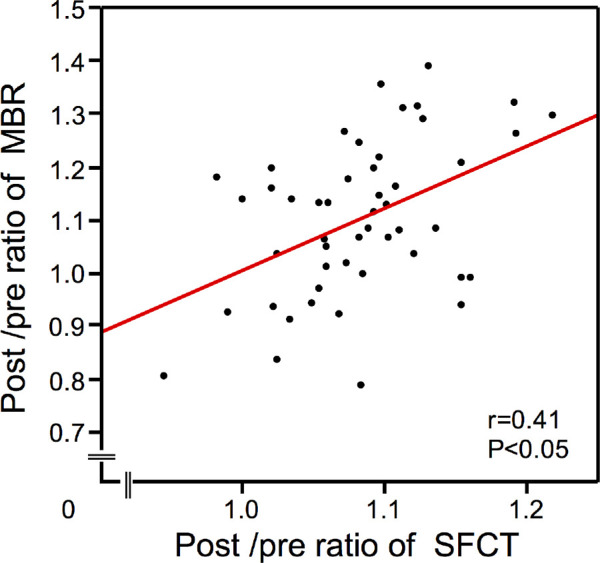
Correlation between the post-/pre-ratio of SFCT and MBR. The correlation between the post-/pre-ratio of SFCT and MBR at 1 week after strabismus surgery is presented. There was a significantly positive correlation between the pre-/post-ratio of SFCT and MBR (*r* = 0.41, *P* < 0.05). Post, postoperative; Pre, preoperative; 1week, one week after surgeries.

The study included 254 eyes from 127 patients aged three years or older who underwent strabismus surgery at Nagoya University Hospital from January 2019 to November 2022 and who consented to the study. Consent for the study was obtained from adult participants and from parents of participants <20 years of age. Eyes with a previous history of strabismus surgery (n = 8), epiretinal membrane surgery (n = 1), macular disease (n = 2), optic nerve atrophy (n = 2), nystagmus (n = 2), transposition surgery for rectus muscle palsy (n = 2), and aborted surgery (n = 2) were excluded. Finally, 133eyes from 80 patients (operated eyes = 116, unoperated eyes = 17) for which data could be obtained were included in the analysis. Table 1 shows the preoperative characteristics, and Table 2 presents the surgical procedures. Figure 1 depicts the representative multimodal images of a patient who underwent lateral rectus muscle recession and medial rectus muscle plication. Figure 2 shows the changes in the post-/pre-ratio of the SFCT, MBR, and VD of the operated eyes during the observation period, and Table 3 shows the median post-/pre-ratio of the measurements at one week, one month, and four months after surgery. As mentioned in the Methods section, MBR is an indicator of choroidal blood flow and VD is an indicator of retinal blood flow. The post-/pre-ratio of SFCT at one week and one month after surgery were significantly higher than those before surgery (*P* < 0.001, *P* = 0.010). However, there was no significant change in the post-/pre-ratio of SFCT at four months after surgery. The post-/pre-ratio of MBR at one week was significantly higher than that before surgery (*P* < 0.001). Nevertheless, the post-/pre-ratio of MBR at one and four months after surgery did not significantly differ. The post-/pre-ratio of VD was significantly lower at one week after surgery (*P* = 0.0016), with no significant change at one and four months after surgery (Fig. 2). Figure 3 depicts the correlation between the post-/pre-ratio of SFCT and the post-/pre-ratio of MBR. The choroidal thickness and choroidal blood flow significantly increased at one week. To validate the association between choroidal thickness and blood flow at that time, the correlation between choroidal thickness and blood flow was examined. Results showed a significantly positive correlation between choroidal thickness and choroidal blood flow at one week after surgery (*r* = 0.41, *P* < 0.05).

**Table 1.** The Preoperative Characteristics of Operated Eyes (n = 116)

Variable	
Age, median (IQR), yrs	13.5 (9–27)
Male	62 (53.4%)
General anesthesia^*^	87 (75.0%)
Refractive value, median (IQR), Diopter	−1.3 (−4 to 0.25)
Preoperative BCVA, median (IQR), Log MAR	0 (0–0)
Preoperative IOP, median (IQR), mmHg	15 (13–18)
SFCT,^†^ median (IQR), µm	258.2 (196.3–315.0)
VD,^‡^ median (IQR), mm^2^/mm^2^	35.8 (34.0–37.0)
MBR,^§^ median (IQR), AU	9.7 (7.8–11)

AU, arbitrary units; BCVA, best-corrected visual acuity; IOP, intraocular pressure; IQR, interquartile range; LogMAR, logarithm of minimal angle of resolution.

* If general anesthesia was not used, surgeries were performed with the subjects under local anesthesia.

† SFCT was measured using enhanced-depth imaging OCT from the retinal pigment epithelium to the choroid sclera boundary.

‡ VD was measured using OCT angiography within a 3  × 3-mm circle centered on the macula, expressed as the area occupied by blood vessels per unit area (mm²/mm²).

§ MBR was measured with laser speckle flowgraphy, representing relative choroidal blood flow by detecting speckle contrast caused by erythrocyte movement.

**Table 2.** Surgical Procedures

Surgical Procedures	Type of Rectus Muscle	Number of Eyes
Rectus muscle recession and IO recession	LR	7
	LR (+ MR plication)	22
	MR	3
	SR	1
	IR	2
	LR + SR	1
	LR + IR	1
Rectus muscle recession	LR	11
	LR (+ MR plication)	28
	MR	7
	MR (+ LR plication)	1
	LR + MR	1
	LR + SO	1
	SR	1
	IR	6
IO recession		22
SO recession		1

IO, inferior oblique muscle; IR, inferior rectus muscle; LR, lateral rectus muscle; MR, medial rectus muscle; SO, superior oblique muscle; SR, superior rectus muscle.

**Table 3.** Median of the Changes in the Post-/Pre-Ratio of SFCT, MBR, and VD in the Eyes Undergoing Strabismus Surgery

	Median Post-/Pre-Ratio		
	SFCT	MBR	VD
1 week after surgery	1.07 (1.02–1.10)	1.07 (0.99–1.19)	0.98 (0.93–1.02)
1 month after surgery	1.02 (0.97–1.06)	1.06 (0.91–1.15)	1.00 (0.96–1.05)
4 months after surgery	0.99 (0.94–1.04)	0.96 (0.87–1.15)	1.00 (0.94–1.03)

**Figure 4.**
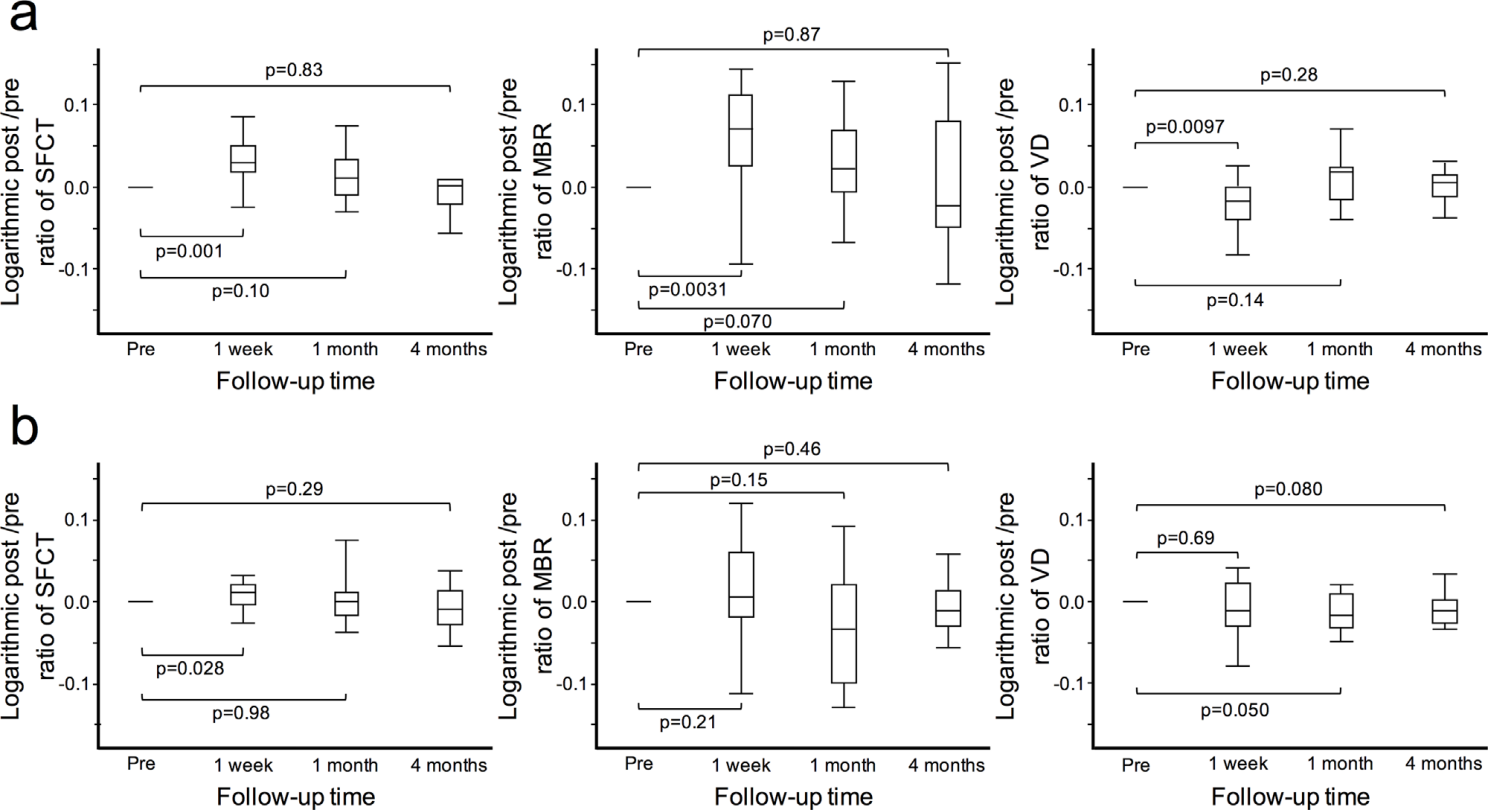
Changes in the post-/pre-ratio of SFCT, MBR, and VD between the operated and unoperated eyes in patients undergoing unilateral eye surgery. (**a**) In the operated eyes, the SFCT increased significantly at one week after surgery (*P* = 0.0010). The choroidal MBR increased significantly at one week after surgery (*P* = 0.0031), and the VD decreased significantly at one week after surgery (*P* = 0.0097). However, none of the measurements changed significantly at four months after surgery. (**b**) In the unoperated eyes, there were no significant changes in SFCT, MBR, or VD at all timepoints. Pre, preoperative; 1 week, one week after surgeries; 1 month, one month after surgeries; 4 months, four months after surgeries.

To determine whether the current changes were attributed to strabismus surgery, the changes in preoperative and postoperative measurements in the operated and unoperated eye in 17 patients who had undergone surgery in one eye were further examined (Fig. 4). In the operated eye, the post-/pre-ratio of SFCT increased significantly at one week after surgery (*P* = 0.0010), with no significant change at one and four months after surgery. The post-/pre-ratio of MBR increased significantly at one week after surgery (*P* = 0.0031), with no significant change at one and four months after surgery. The post-/pre-ratio of VD also decreased significantly at one week (*P* = 0.0097), with no significant change at one and four months after surgery. The operated eye exhibited similar changes to the overall results. Conversely, in the unoperated eyes, there were no significant changes in SFCT, MBR, or VD at all timepoints.

**Figure 5.**
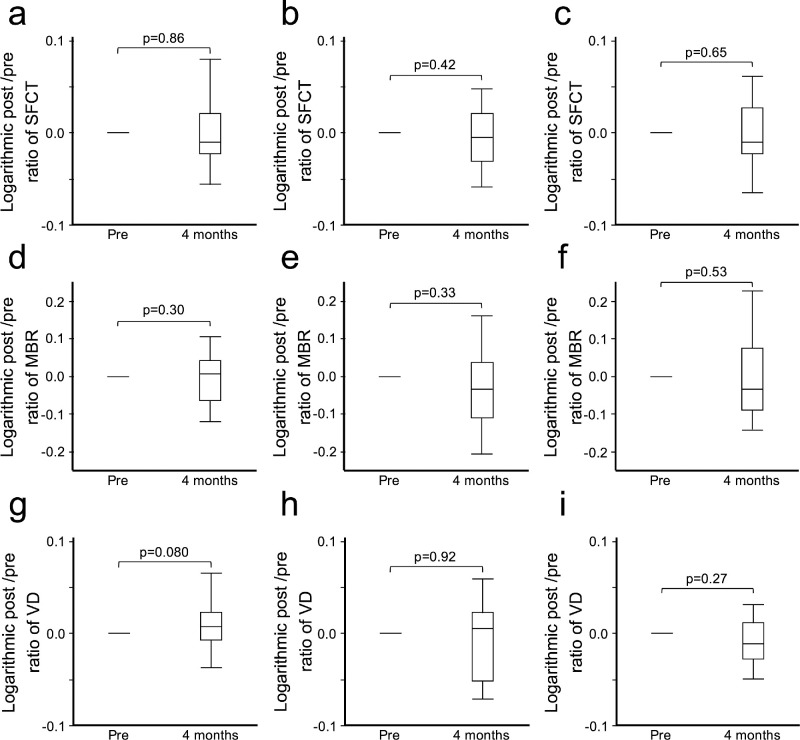
Change in the pre-/post-ratio of SFCT (**a**–**c**), MBR (**d**–**f**), and VD (**g**–**i**) according to different surgical procedures. (**a**, **d**, **g**) The group who underwent LR recession and MR plication did not exhibit significant changes in the pre-/post-ratio of SFCT, MBR, and VD at four months after surgery. (**b**, **e**, **h**) The group who underwent IO recession alone did not exhibit significant changes in the pre-/post-ratio of SFCT, MBR, and VD at four months after surgery. (**c**, **f**, **i**) The group who underwent LR recession, MR plication, and IO recession did not exhibit significant changes in the pre-/post-ratio of SFCT, MBR, and VD at four months after surgery. IO, inferior oblique muscle; LR, lateral rectus muscle; MR, medial rectus muscle; Pre, preoperative; 1 week, one week after surgeries; 1 month, one month after surgeries; 4 months, four months after surgeries.

Because this study included several surgical procedures, the effect of postoperative retina and choroid might differ based on the surgical procedure. Therefore the patients were divided into groups according to surgical procedure and the changes in retina and choriod at four months after surgery in each group were examined (Fig. 5). However, no significant changes were observed in the analysis groups based on the surgical procedure.

## Discussion

In this study, we examined whether strabismus surgery with extraocular muscle recession can reduce anterior ocular and choroidal blood flow. Few studies have examined the effect of strabismus surgery on posterior ocular blood flow, particularly retinal and choroidal blood flow. However, with the use of newly introduced techniques (such as OCT, OCTA, and LSFG), the retinal and choroidal thickness and blood flow can now be evaluated independently.^14^^,^^25^ To the best of our knowledge, this is the first report on quantitative changes in choroidal blood flow measured by LSFG following strabismus surgery.

In the current study, the SFCT and choroidal blood flow increased significantly at one week after surgery. Several studies have investigated the effects of strabismus surgery on the choroid; Inan et al.^26^ and Yetkin et al.^27^ provided basic insight into postoperative changes by showing a significant decrease in SFCT after strabismus surgery. However, choroidal blood flow was not quantitatively assessed. Xiao et al.^28^ used OCTA to quantitatively assess changes in choroidal thickness and choroidal blood flow after horizontal rectus muscle surgery, which contributed significantly to our understanding on blood flow changes. OCTA is suitable for assessing structural changes in choroidal details. However, it has a limitation. Particularly, it cannot assess blood flow in the entire choroid postoperatively. This study used LSFG to quantitatively validate changes in the whole choroidal blood flow postoperatively. The association between choroidal thickness and blood flow remains controversial, and not all studies have always shown a correlation between the two.^29^^,^^30^ Therefore choroidal thickness and blood flow must be evaluated independently, and this is what we focused on in this study. A positive correlation was found between SFCT and choroidal blood flow at one week after surgery. Margraf et al.^31^ have revealed how surgical trauma triggers the release of damage-associated molecular patterns, which activate immune responses and promote endothelial dysfunction. This leads to increased vascular permeability, leukocyte recruitment, and local tissue swelling. These processes disrupt normal vascular dynamics, and they may explain the increase in choroidal blood flow and thickness postsurgery.^28^^,^^31^ The differences between the changes in the operated eyes and unoperated eyes of patients who had undergone unilateral strabismus surgery indicated that the abovementioned changes were caused by the strabismus surgery.

In the long-term postoperative period, the SFCT and choroidal blood flow decreased at four months after surgery; the difference was not significant. The choroidal blood flow might differ based on the surgical techniques used. Hence, the patients were classified according to surgical techniques, and the choroidal blood flow was examined at four months after surgery. However, the results showed no significant differences in the choroidal blood flow in any of groups. These findings suggest that the impact of surgical combinations on choroidal blood flow may be less evident than initially anticipated. Nevertheless, the short follow-up period (four months) and small sample size might have reduced the statistical power of the analysis. These results emphasize the need for more comprehensive investigations on the effects of surgical combinations on posterior ocular blood flow. Thus future studies should focus on long-term follow-up, consider individual variability, and involve multicenter collaborations to increase sample size and ensure robust conclusions. Previous studies have shown that reduced choroidal blood flow may cause retinal ischemia, age-related macular degeneration, and ocular axis elongation.^12^^,^^32^ Thus reduced choroidal blood flow after strabismus surgery may lead to long-term complications. Strabismus surgery is often performed on children. Thus studies focusing on the long-term prognosis of patients undergoing strabismus surgery should be performed.

Contrary to choroidal blood flow, retinal blood flow decreased at one week after surgery. The posterior ciliary artery (PCA) is responsible for blood flow to the choroid. Furthermore, PCA hemodynamics is correlated with choroidal blood flow, and retinal blood flow with central retinal artery blood flow.^33^ Because the PCA and central retinal artery are branches of the ocular artery, an increase in PCA blood flow may result in a decrease in central retinal artery blood flow. Therefore we hypothesized that choroidal and PCA blood flow increased at one week after surgery, resulting in a decrease in central retinal artery and retinal blood flow.

This study had several limitations. First, it was performed at a single center, and only standard procedures performed at our institution were examined. Therefore the effects of procedures performed at other institutions such as recession of two or more rectus muscles and IO myectomy on the retina and choroid were not comprehensively examined. Second, in this study, two surgeons performed the surgery. However, the influence of surgeon differences was not considered. The scanning range of the OCTA used in this study was 3  × 3 mm, which is smaller than that used in glaucoma research and other studies. This size is used to detect minute changes in the macula and to improve measurement accuracy in studies on children and patients with strabismus who have fixation that was difficult to stabilize. However, scanning a wider area may be more effective for evaluating changes in blood flow. Finally, the number of cases in this study was small. Therefore a long-term follow-up study with a larger number of patients should be performed to ensure the generalizability of the study results.

In summary, strabismus surgery decreased retinal blood flow and increased choroidal thickness and blood flow in the early postoperative period. However, no significant changes were observed in the long term compared with the preoperative period.
